# Hashimoto Thyroiditis and Progression of Papillary Thyroid Cancer: 10-Year Retrospective Cohort Study

**DOI:** 10.2196/80535

**Published:** 2026-01-06

**Authors:** Xin Li, Xiangyun Yao, Rui Shan, Fang Mei, Shibing Song, Bangkai Sun, Chunhui Yuan, Zheng Liu

**Affiliations:** 1 Department of General Surgery, Peking University Third Hospital, Beijing, China; 2 Department of Ultrasound, Peking University Third Hospital, Beijing, China; 3 Department of Maternal and Child Health, School of Public Health, Peking University, 38 Xueyuan Road , Haidian District, Beijing, 100191, China, 86 82801222; 4 Department of Pathology, Peking University Third Hospital, Beijing, China; 5 School of Basic Medical Sciences, Peking University Health Science Center, Beijing, China; 6 Information Management and Big Data Center, Peking University Third Hospital, Beijing, China

**Keywords:** papillary thyroid cancer, Hashimoto thyroiditis, progression, retrospective cohort., progression-free survival, lymph node

## Abstract

**Background:**

In recent years, the global incidence of thyroid cancer has been increasing.

**Objective:**

This study aimed to examine the association between Hashimoto thyroiditis (HT) and papillary thyroid cancer (PTC) progression under active surveillance (AS).

**Methods:**

Our retrospective study was conducted at Peking University Third Hospital and included 203 patients with PTC who underwent AS for ≥6 months before surgery. Patients were first categorized into 2 groups: the HT group (n=90) and the non-HT group (n=113). Cox proportional hazards models were then used to evaluate the association between HT and PTC progression during AS, adjusting for age, sex, baseline tumor size, BMI, pregnancy status, number of tumor foci, and thyroid-stimulating hormone level. Subgroup analyses stratified by the 6 covariates mentioned above were also applied to explore the potential effect modification.

**Results:**

No significant difference was observed between the HT and non-HT groups in PTC progression-free survival (hazard ratio [HR] 1.11, 95% CI 0.61‐1.99; *P*=.74), tumor enlargement-free survival (HR 1.02, 95% CI 0.56‐1.86; *P*=.95), or lymph node metastasis-free survival (HR 1.76, 95% CI 0.31‐10.12; *P*=.52). Subgroup analyses revealed a potential interaction between HT and BMI. Among patients who were overweight or obese (BMI >24 kg/m²), HT was significantly associated with an increased risk of disease progression (HR 6.32, 95% CI 1.84‐21.69; *P*=.003), while among patients with BMI ≤24 kg/m^2^, no association between HT and progression risk was observed (*P*=.01).

**Conclusions:**

We found no evidence of association between HT and PTC progression during AS. However, the relationship between HT and PTC progression may be modified by overweight or obesity status.

## Introduction

Thyroid cancer is not only the most common malignant tumor of the head and neck but also the most prevalent endocrine malignancy. In recent years, the global incidence of thyroid cancer has been steadily increasing. Between 1990 and 2020, the number of newly diagnosed cases worldwide rose from 95,000 to 586,000 [1-3]. In China, the age-standardized incidence rate increased from 3.21 per 100,000 in 2005 to 24.64 per 100,000 in 2022 [4,5]. Clinically, papillary thyroid cancer (PTC) is the most frequently encountered, representing approximately 80% of all thyroid cancer cases worldwide [6].

As understanding of PTC has evolved, active surveillance (AS) has been increasingly adopted by Japan and Korea as part of a comprehensive management approach for low-risk PTC [7,8]. The US and Chinese guidelines also consider AS an acceptable treatment approach [9,10]. AS is an emerging management strategy that offers a more conservative alternative to immediate surgery [9]. It involves active monitoring of the patient’s condition without initiating surgical treatment unless there is evidence of tumor progression [11]. Active monitoring primarily refers to performing neck ultrasound every 6 months for the first 1 to 2 years, followed by annual examinations. Despite growing interest in AS for low-risk PTC, there is still no clear consensus on which patients should undergo immediate surgery and which are appropriate candidates for AS.

Hashimoto thyroiditis (HT) is an autoimmune disorder characterized by immune-mediated destruction of thyroid cells through both cellular and humoral mechanisms [12]. The incidence of HT has been increasing steadily over the years, and its coexistence with PTC is relatively common [13-15]. Some studies have suggested that HT may be a risk factor for the development of PTC, while others have indicated that HT could be a protective factor against postoperative recurrence of PTC [13,14,16].

Nevertheless, the vast majority of existing research has focused solely on the postoperative recurrence, overlooking the increasingly prevalent management strategy of AS in recent years. Currently, there is limited evidence on the association between HT and PTC progression during AS. Importantly, the risk factors for postoperative recurrence and those for disease progression under AS may differ substantially [9,17]. Identifying the risk factors for tumor progression during AS is crucial for selecting appropriate candidates, helping to avoid both undertreatment and overtreatment [9]. Moreover, patients with HT tend to be younger and more likely to be female compared to those without HT [14]; both sex and age have been independently associated with disease progression [18,19]. Therefore, it is essential to investigate the role of HT in PTC progression while carefully accounting for potential confounders.

Two reviews have investigated the role of HT in PTC progression [3,13]; however, they have several notable limitations:

1. Lack of stringent inclusion and exclusion criteria: most of the included studies focused on patients who underwent surgery, providing limited insight into the role of HT during AS.
2. Failure to account for confounding factors: key confounders such as sex and age were not adequately considered. For example, the observed association between HT and favorable prognosis in some studies may be attributed to the higher proportion of female patients in the HT group.
3. Unclear definition of outcome measures: these reviews did not clearly distinguish between the role of HT in tumor progression during AS and its effect on recurrence after surgery. This distinction is important, as the extent of surgery may differ for patients with HT and may itself act as a confounding factor in recurrence outcomes.
4. Lack of quantitative synthesis: one of the reviews did not include a quantitative meta-analysis, limiting the ability to draw robust conclusions [3].

Therefore, we investigated the relationship between HT and progression-free survival of PTC under AS—including tumor enlargement and lymph node metastasis (LNM)—while controlling for multiple potential confounding factors.

## Methods

### Ethical Considerations

The study was approved by the Medical Ethics Committee of Peking University Third Hospital (ethical project number: IRB00006761-M2022721). The requirement for informed consent was waived by the Ethics Committee due to the retrospective nature of the study. All patient data were deidentified and anonymized prior to analysis to ensure the privacy of individuals.

### Study Population

The Electronic Medical Record system of Peking University Third Hospital was searched to retrieve cases of thyroid surgery with a subsequent diagnosis of PTC, covering the period from January 2012 to September 2022.

The AS strategy was routinely implemented at our institution beginning in 2015. Since then, all patients diagnosed with low-risk PTC have been introduced to the AS option during outpatient consultations. Following comprehensive counseling by clinicians regarding the risks associated with thyroid surgery, the potential need for thyroid hormone replacement therapy, and the possibility of disease progression during AS, patients make their decision based on individual circumstances—such as pregnancy plans and overall health status—on whether to pursue AS. Patients independently decided whether to undergo AS at our center.

Based on previous studies [20-23] and domain knowledge, the inclusion criteria for the study population were as follows: (1) patients with a pathological diagnosis of PTC in surgical paraffin-embedded specimens; this criterion was feasible because the specimen acquisition method is consistently documented in all pathology reports; (2) patients who underwent at least two thyroid ultrasound examinations prior to surgery at our center; and (3) patients who underwent ≥6 months of preoperative surveillance; (4) patients without surgical contraindications. Patients who presented with LNM or extrathyroidal extension (ETE) at baseline were excluded, as AS is generally not recommended for individuals with evidence of metastasis. For patients with multifocal PTC, only the lesion with the largest mean tumor diameter was considered for analysis [24]. Ultimately, based on the presence or absence of HT, the study population was divided into the HT group and the non-HT group.

Following relevant guidelines and previous studies [14,25], the criteria for diagnosing HT were defined as meeting at least one of the following conditions: (1) pathological evidence of HT in the peritumoral thyroid tissue on the postoperative pathology report or (2) thyroid function test results within 30 days before surgery showing thyroglobulin antibody ≥60 U/mL or ≥4.5 IU/mL, or thyroid peroxidase antibody ≥60 U/mL or ≥34 IU/mL.

### Progression During Surveillance

The primary outcome of this study was progression-free survival, defined as the time from baseline to the progression of PTC. Tumor progression was determined based on either of the following criteria [26] : (1) tumor enlargement, defined as an increase of ≥3 mm in any tumor diameter or (2) newly detected LNM during surveillance. In cases of suspected progression, original pathological, ultrasonographic, and surgical records were independently reviewed by senior clinicians and investigators to ensure accurate determination of disease progression.

### Data Extraction

All authors involved in data entry for this study received standardized and targeted training to ensure consistency and comparability of the collected data. The demographic characteristics, imaging examinations, laboratory results, and pathological data extracted from the Electronic Medical Record system were entered and consolidated using Epidata software. To minimize subjective bias during data extraction, patient information was anonymized. Quality control was implemented at multiple stages. Initial and midterm audits were independently conducted by investigators: for each data entry personnel, 20 records were randomly selected and cross-checked against the original source documents to verify accuracy. After data entry was completed, a final spot-check review was performed by a senior physician to ensure the overall accuracy and reliability of the dataset. Data filtering was implemented in R (R Core Team) language according to the predetermined enrollment criteria.

### Statistical Analyses

First, we compared baseline characteristics between the HT group and the non-HT group. For categorical variables, frequencies and percentages were reported, and differences between groups were assessed using the Pearson chi-square test or the Fisher exact test when the expected cell count was ≤5. For continuous variables, normality was tested using the Shapiro-Wilk test. Since the data did not follow a normal distribution, group differences were analyzed using the Mann-Whitney *U* test.

Next, to examine the association between HT and progression-free survival, we first tested the proportional hazards assumption using Schoenfeld residuals. We then constructed a multivariable Cox proportional hazards regression model, adjusting for potential confounders, including age, sex, baseline maximal tumor diameter, BMI, pregnancy status, number of tumor foci, and thyroid-stimulating hormone (TSH) level [27,28]. To visually illustrate the survival differences between the HT and non-HT groups, adjusted Kaplan-Meier PTC progression-free survival curves were plotted.

Further subgroup analyses were conducted based on median age (≥36 or <36 y), median TSH level (>1.72 or ≤1.72 μIU/mL), sex (male or female), baseline tumor size (the maximum diameter >1 or ≤1 cm), number of tumor foci (single or multiple), BMI (>24.0 or ≤24.0 kg/m^2^), and pregnancy status (pregnant or not). We evaluated the modifying effects of these variables on the association between HT and progression-free survival using interaction terms (subgroup variable × HT/non-HT group) within the Cox regression model. In the subgroup analyses, covariates other than the stratifying variable were adjusted for accordingly. For example, when stratifying by sex, adjustments were made for age, TSH level, baseline tumor size, number of tumor foci, BMI, and pregnancy status. Similarly, when stratifying by age, adjustments were made for sex, TSH level, baseline tumor size, number of tumor foci, BMI, and pregnancy status.

All statistical analyses were performed using R software (version 4.4.3; R Foundation for Statistical Computing). A 2-sided *P* value of <.05 was considered statistically significant.

## Results

### Characteristics of the Study Population

**Figure 1.**
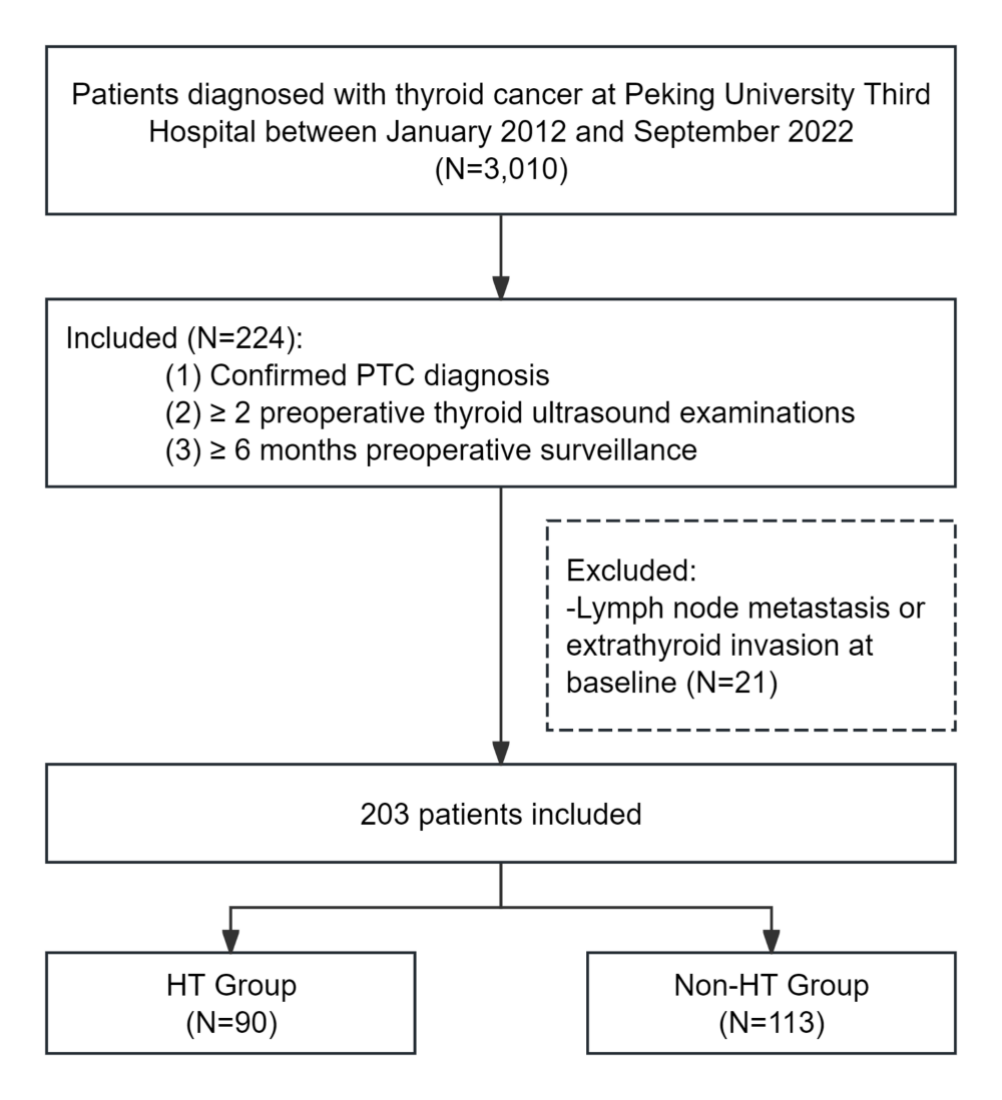
Study flow diagram of the inclusion. HT: Hashimoto thyroiditis; PTC: papillary thyroid carcinoma.

A total of 203 patients with PTC under AS were included in the study. The median age was 36 (IQR 30-42) years, and 187 (92%) patients were female. The median preoperative surveillance duration was 1.45 (IQR 0.89-2.60) years. Patients were categorized into the HT group (n=90) and the non-HT group (n=113). The study flow diagram is presented in Figure 1, and the baseline characteristics of both groups are summarized in Table 1. Significant differences in age and sex were observed between the 2 groups (both *P*<.001). Table 2 shows the progression status of the study population.

**Table 1.** Baseline characteristics of the study population.

Characteristics	HT^a^ group (n=90)	Non-HT group (n=113)	*P* value
Age (y), median (IQR)	34.5 (30.0-39.8)	39.0 (31.0-45.0)	<.001^b^
Baseline maximal tumor diameter (cm), median (IQR)	0.7 (0.6-1.1)	0.8 (0.6-1.2)	.41^b^
Surveillance duration (y), median (IQR)	1.53 (0.83-2.74)	1.44 (0.93-2.49)	.86^b^
Sex, n (%)			<.001^c^
Female	89 (98.9)	98 (86.7)	
BMI (kg/m^2^), median (IQR)	23.4 (21.1-25.4)	23.4 (21.5-26.9)	.42^b^
Pregnancy status, n (%)			.47^d^
Yes	8 (8.9)	6 (5.3)	
Number of tumor foci, n (%)			.06^d^
Single	53 (58.9)	82 (72.6)	
Multiple	37 (41.1)	31 (27.4)	
Thyroid-stimulating hormone (TSH) level (μIU/mL), median (IQR)^e^	1.83 (1.12-2.56)	1.64 (1.26-2.18)	.29^b^

a HT: Hashimoto thyroiditis.

b Mann-Whitney *U* test.

c Fisher exact test.

d Pearson *χ*2 test with Yates’ continuity correction.

e TSH levels were missing in 93 patients during the observation period.

**Table 2.** Progression status of the study population.

Progression type	HT^a^ group (n=90)	Non-HT group (n=113)	*P* value
PTC^b^ progression, n (%)			
Yes	30 (33.3)	32 (28.3)	.54^c^
Tumor enlargement, n (%)			
Yes	28 (31.1)	31 (27.4)	.68^c^
Lymph node metastasis, n (%)			
Yes	6 (6.7)	3 (2.7)	.19^d^

a HT: Hashimoto thyroiditis.

b PTC: papillary thyroid carcinoma.

c Pearson *χ*2 test with Yates’ continuity correction.

d Fisher exact test.

### Association Between HT and Progression-Free Survival

The Schoenfeld residuals test indicated that our data met the proportional hazards assumption (*P*>.05). During the surveillance period, 63 (31.0%) patients experienced progression, with 30 (33.3%) patients in the HT group and 33 (29.2%) patients in the non-HT group. Among the 63 patients who progressed, 6 experienced both tumor enlargement and LNM, 54 had tumor enlargement only, and 3 had LNM only.

In the multivariable Cox regression analysis, after adjusting for age, sex, and baseline mean tumor diameter, no significant difference in PTC progression-free survival was observed between the HT and non-HT groups (hazard ratio [HR] 1.11, 95% CI 0.61‐1.99; *P*=.74). Similarly, no significant differences were found in tumor enlargement-free survival (HR 1.02, 95% CI 0.56‐1.86; *P*=.95) or LNM-free survival (HR 1.76, 95% CI 0.31‐10.12; *P*=.52; Table 3).

**Figure 2.**
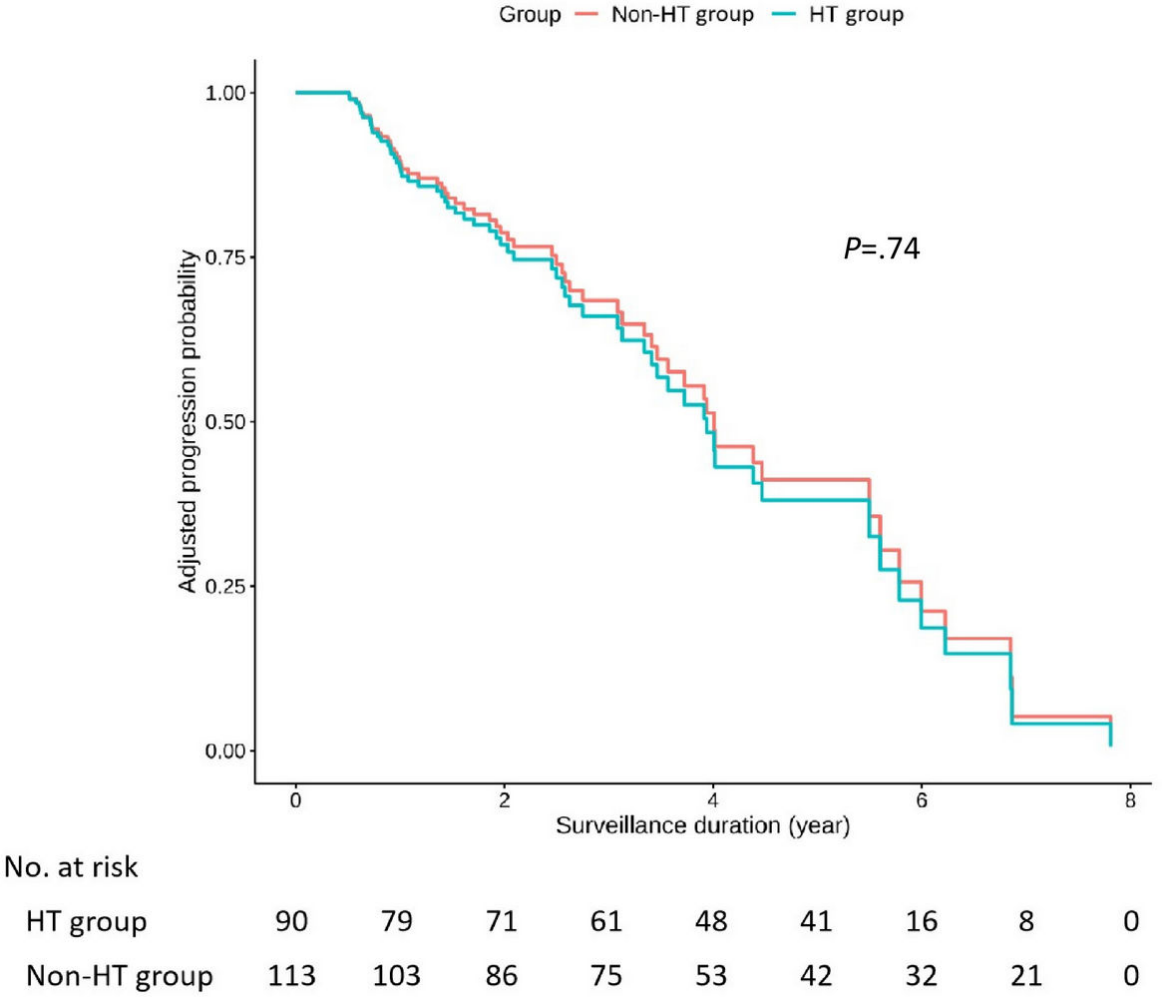
Kaplan-Meier papillary thyroid carcinoma progression-free survival curves. Adjust for age, sex, baseline maximal tumor diameter, BMI, pregnancy status, number of tumor foci, and thyroid-stimulating hormone level. HT: Hashimoto thyroiditis.

The Kaplan-Meier curves for PTC progression-free survival revealed that the survival curves of the HT and non-HT groups were nearly identical, indicating no significant difference between the 2 groups (Figure 2).

**Table 3.** Association between HT^a^ and progression-free survival.

Outcomes	HR^b^^c^ (95% CI)	*P* value
PTC^d^ progression	1.11 (0.61-1.99)	.74
Tumor enlargement	1.02 (0.56-1.86)	.95
Lymph node metastasis	1.76 (0.31-10.12)	.52

a HT: Hashimoto thyroiditis.

b HR: hazard ratio.

c Adjust for age, sex, baseline maximal tumor diameter, BMI, pregnancy status, number of tumor foci, and thyroid-stimulating hormone level.

d PTC: papillary thyroid carcinoma.

### Sensitivity Analyses

In the sensitivity analyses, no significant difference in PTC progression-free survival was observed between the HT and non-HT groups (HR 1.23, 95% CI 0.60‐2.53; *P*=.58). Similarly, no significant differences were found in tumor enlargement–free survival (HR 1.08, 95% CI 0.51‐2.28; *P*=.84) or LNM-free survival (HR 2.44, 95% CI 0.23‐25.23; *P*=.46). The findings from the sensitivity analyses were consistent with those of the primary analyses, suggesting the robustness of the results.

### Subgroup Analyses

**Figure 3.**
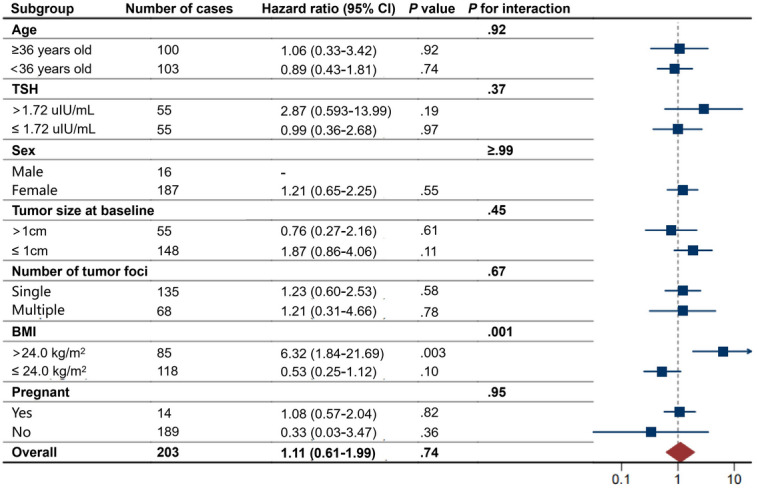
Subgroup analysis of the association between Hashimoto thyroiditis (HT) and progression-free survival. TSH levels were missing in 93 patients during the observation period. In the male subgroup, only 1 patient had HT, and no progression was observed in this case; therefore, an estimate could not be provided. TSH: thyroid-stimulating hormone.

Figure 3 shows the forest plot of subgroup analyses. There was no significant difference in the association between HT and progression-free survival across subgroups classified by median age (36 y), median TSH level (1.72 μIU/mL), sex, baseline tumor size (the maximum diameter >1 or ≤1 cm), number of tumor foci, or pregnancy status (*P*_interaction_>.05). Notably, we found a potential interaction between HT and BMI (*P*_interaction_<.01). Among patients who were overweight or obese (BMI >24 kg/m^2^), HT was associated with a significantly higher risk of progression (HR 6.32, 95% CI 1.84‐21.69; *P*=.003). In contrast, no association between HT and progression risk was observed in patients with BMI ≤24 kg/m^2^ (*P*=.10).

## Discussion

### Summary of the Findings

Over a median surveillance period of 1.45 years, our analysis demonstrated no significant association between HT and PTC progression under AS. This finding was consistent for both tumor enlargement and LNM. The results remained consistent in the sensitivity analysis. In the subgroup analyses, no significant association between HT and progression-free survival was found across strata defined by age, TSH level, sex, baseline tumor size, number of tumor foci, or pregnancy status. However, a potential interaction between HT and BMI was identified. Among patients who were overweight or obese (BMI >24 kg/m²), HT was significantly associated with an increased risk of progression.

These results suggest that HT is not independently associated with PTC progression during AS in the general population. However, our subgroup analysis revealed a potential interaction between HT and BMI, suggesting that the association between HT and PTC progression may be modified by overweight or obesity status. Overweight is an increasingly recognized clinical condition. The association between obesity and ETE, tumor multifocality, larger tumor size, as well as LNM remains controversial. However, it has been established that a positive correlation exists between elevated BMI and the presence of the BRAFV600E mutation [29]. Although HT has been proven to be a factor for a better prognosis, this study confirms that, compared to other clinical indicators, an association between HT and PTC progression can be observed specifically within the overweight subgroup in this study. Although the current understanding of the mechanisms underlying the interaction between obesity and HT in PTC progression remains unclear, we speculate that their association may involve the following aspects: (1) HT is associated with a lower BRAF V600E mutation rate [14], while obesity is associated with a higher BRAF V600E mutation rate [30,31]. When both conditions coexist, their opposing effects on gene mutation might offset each other. (2) HT is characterized by lymphocytic infiltration in thyroid tissue. Patients with HT and PTC have been observed to have reduced Tregs and increased interleukin 10 secretion [32]. Conversely, obesity induces Treg expansion and elevated interleukin 10 [33], which may indirectly promote tumor immune escape by suppressing the cytotoxic functions of CD8^+^ T cells and natural killer cells, thereby counteracting the influence of HT on PTC. (3) Regarding metabolism, in the state of obesity, increased levels of free fatty acids provide more energy for tumor cells.

Further studies with larger cohorts and mechanistic investigations were warranted to validate and elucidate this interaction.

### Comparison With Previous Studies

Previous studies have provided substantial evidence supporting a potential association between HT and PTC prognosis. For example, Marotta et al [34] conducted a multicenter retrospective cohort study in Italy involving 301 patients with PTC, of whom 42.5% had coexisting HT and reported significantly longer recurrence-free survival in those with HT. Similarly, Xu et al [14] performed a single-center retrospective cohort study in China involving 9210 patients with PTC, with a 19% prevalence of coexistent HT. Xu et al also suggested that HT was associated with better PTC prognosis.

Nevertheless, the association between HT and postoperative recurrence may differ substantially from its relationship with disease progression under AS. Yet, this distinction has been largely overlooked, as few studies have specifically investigated the impact of HT on PTC progression during AS. In a Korean cohort of 699 patients with PTC managed with AS, Lee et al [17] reported that tumor progression was associated with diffuse thyroid disease (DTD) as detected by ultrasound, with a 2.3-fold increased risk of progression in patients with HT compared to those without. Interestingly, although our study did not find a statistically significant association between HT and PTC progression under AS in the general population, we observed a 6.32-fold increased risk of progression associated with HT in patients who were overweight or obese. It is important to highlight that our study design differs from that of Lee et al [17]. First, HT represents only one type of DTD, which also includes other conditions such as Graves’ disease and simple goiter. Second, according to Lee et al [17], DTD was diagnosed based on preoperative ultrasound examinations, while in our study, the diagnosis of HT was primarily based on postoperative pathological confirmation and preoperative serum antibody levels measured within 30 days before surgery.

### Limitations and Strengths

Our study has several strengths. First, we applied strict eligibility criteria, including only patients who had undergone AS for more than 6 months preoperatively, to ensure that sufficient follow-up time was available for detecting clinically meaningful changes. The relatively low enrollment proportion is attributable to 2 main factors: (1) AS has only been widely adopted in recent years, and many preoperative cases did not undergo this protocol. (2) Some nonlocal patients or those seeking examinations at other hospitals underwent only a single ultrasound at our institution prior to surgery, making them largely ineligible. We also excluded patients with baseline LNM or ETE to reduce confounding by indication. Second, we employed Cox proportional hazards models to fully utilize time-to-event data, providing more robust estimates. Third, all outcome events were individually reviewed by experienced clinicians to ensure the accuracy of progression assessment. Fourth, beyond the overall analysis, we examined the association between HT and specific types of progression (including tumor enlargement and LNM) and conducted sensitivity analyses to assess the robustness of our findings. Furthermore, we investigated potential interactions and associations across various patient subgroups.

Nevertheless, our results should be interpreted with caution. First, this was a retrospective study based on data extracted from electronic medical records. We plan to conduct a well-designed, prospectively followed cohort study with standardized data collection to validate our findings. Second, in clinical practice, the decision to undergo AS is influenced not only by the biological risk of tumor progression but also by patient preferences. For instance, patients with significant anxiety may opt for surgery even if their clinical condition qualifies for observation. Such preference-based decisions could introduce selection bias in our study population. Third, as a single-center study, the generalizability of our findings may be limited. Fourth, the limited sample size restricted our adjustment to only a constrained set of confounders. Future studies with larger cohorts are needed to control for a broader range of potential confounding factors. Fifth, the relatively short follow-up period may have limited our ability to detect long-term disease progression dynamics. In several instances, surgical intervention occurred before tumor progression could be observed, which could introduce observational time bias and potentially affect the accuracy of outcome assessments.

These findings should be regarded as hypothesis-generating and may help inform the design of future prospective studies with longer surveillance and more comprehensive data collection.

### Clinical Implications

Given the intensive resources required to conduct prospective cohort studies on AS, there are currently very few studies addressing this issue globally. Our study provides a foundation for future research and has important implications for clinical practice. With a methodologically sound design, we contribute relatively high-quality evidence suggesting that, in general, patients with PTC with coexisting HT who meet clinical criteria for AS do not face an increased risk of disease progression. However, among patients with HT who are overweight or obese (BMI >24 kg/m²), particular attention should be paid to a potentially elevated risk of PTC progression.

### Conclusion

The findings of our retrospective cohort study implied that for patients with PTC, the presence of HT alone might not be a major factor in determining eligibility for AS versus immediate surgery, except in cases where the patients were overweight or obese. We advocate for future prospective cohort studies on AS to validate these findings before they are applied to clinical decision-making.

## Abbreviations

AS: active surveillance
DTD: diffuse thyroid disease
ETE: extrathyroidal extension
HR: hazard ratio
HT: Hashimoto thyroiditis
LNM: lymph node metastasis
PTC: papillary thyroid cancer
TSH: thyroid-stimulating hormone
